# Piezoacoustics for precision control of electrons floating on helium

**DOI:** 10.1038/s41467-021-24452-7

**Published:** 2021-07-06

**Authors:** H. Byeon, K. Nasyedkin, J. R. Lane, N. R. Beysengulov, L. Zhang, R. Loloee, J. Pollanen

**Affiliations:** Department of Physics and Astronomy, Michigan State University, East Lansing, MI USA

**Keywords:** Condensed-matter physics, Quantum information

## Abstract

Piezoelectric surface acoustic waves (SAWs) are powerful for investigating and controlling elementary and collective excitations in condensed matter. In semiconductor two-dimensional electron systems SAWs have been used to reveal the spatial and temporal structure of electronic states, produce quantized charge pumping, and transfer quantum information. In contrast to semiconductors, electrons trapped above the surface of superfluid helium form an ultra-high mobility, two-dimensional electron system home to strongly-interacting Coulomb liquid and solid states, which exhibit non-trivial spatial structure and temporal dynamics prime for SAW-based experiments. Here we report on the coupling of electrons on helium to an evanescent piezoelectric SAW. We demonstrate precision acoustoelectric transport of as little as ~0.01% of the electrons, opening the door to future quantized charge pumping experiments. We also show SAWs are a route to investigating the high-frequency dynamical response, and relaxational processes, of collective excitations of the electronic liquid and solid phases of electrons on helium.

## Abstract (web summary)

Hybrid devices based on electrons on helium may find application in quantum devices. Here the authors demonstrate surface acoustic wave driven acoustoelectric transport of electrons on superfluid helium.

## Introduction

The surface of superfluid helium at low temperatures is a fantastically pristine substrate without the defects that are unavoidable in almost all other material systems. Electrons placed near this superfluid substrate are attracted to it and float ~10 nm above the surface, forming a unique two-dimensional electron system (2DES) with the highest electron mobility in condensed matter^1^. Electrons on helium are quantum non-degenerate, but not classical, with quantum effects influencing the many-electron transport in high magnetic field^2^ as well as the single-electron degrees of freedom.

Strong repulsive forces between electrons in this system conspire to produce electronic fluid^3^ or crystallized electronic solid states^4^ exhibiting exotic high-frequency dynamical response^5–10^, which is typically investigated via free-space coupling between the electrons and radio-frequency or microwave fields. Surface acoustic wave (SAW) based techniques are a promising complementary probe for investigating these high-frequency collective phenomena. The wavelength of a SAW can be made comparable to the inter-electron spacing in this system, opening up SAW-based scattering measurements of the Wigner solid state of electrons on helium^11^ or engineering acoustic lattices^12^ for investigating electron crystallization in the presence of a commensurate trapping potential^13^.

The superfluid substrate is also predicted to facilitate slow electron decoherence, which has attracted interest to electrons on helium as a candidate for quantum information processing^9,14–18^. In this context, recent circuit quantum electrodynamic (cQED) experiments have studied the motion of a single electron on helium coupled to a microwave resonator^18^. Additionally, advances in micro- and nano-fabrication techniques allow for precision control of electrons on helium in confined geometries and mesoscopic devices. Single-electron detection has been experimentally achieved^19^ along with stable, high-fidelity, electron transfer along gated CCD arrays^20^. Point-contact devices^21^, mesoscopic field-effect transistors (FETs)^22,23^, and quasi-1D microchannels^24^ have demonstrated a rich variety of precision transport capabilities in this 2DES on helium. To ultimately realize coherent control of single electrons will likely require devices that bring together multiple of these experimental advances. Given their broad success when coupled with semiconductor systems^25–32^ and quantum circuits^33^, it is natural to ask if SAW techniques could be employed to manipulate electrons on helium and added to the toolkit for precision control of this unique low-dimensional electron system.

Surface acoustic waves on piezoelectric crystals resemble microscopic earthquake-like excitations accompanied by electric fields localized to a region approximately one wavelength above and below the crystal surface. These co-propagating electric fields interact with mobile charges located in close proximity to the piezoelectric surface wave. Since these waves propagate with a speed of several thousand meters per second standard optical and electron beam fabrication techniques are routinely used to create SAW devices with frequencies ranging from 100’s of MHz to several GHz. Of particular interest is the transfer of momentum from a traveling SAW to nearby charges to produce controlled charge pumping^34–37^. In this case the SAW induces a traveling oscillating piezoelectric field that can trap individual electrons, which then propagate in the local minima of the traveling SAW potential (see Supplemental Information Section 2 for a detailed description of the microscopic interaction between the SAW and the system of electrons on helium). Theoretically the interaction of SAWs with electrons on helium was proposed and investigated in Wilen^38^, but to our knowledge no acoustoelectric experiments have been demonstrated with electrons on helium until now.

In this manuscript we report on experiments coupling electrons on superfluid helium-4 to a piezoacoustic SAW-field. The results demonstrate a key step, namely that SAW methods are compatible with electrons on helium and can be used to produce controlled acoustoelectric charge transport. This work paves the way for a class of SAW-based experiments ranging from fundamental studies of the structure of the electron system and its collective excitations to possible hybrid systems coupling small numbers of electrons to quantum acoustic devices^39,40^.

## Results and discussion

**Fig. 1:**
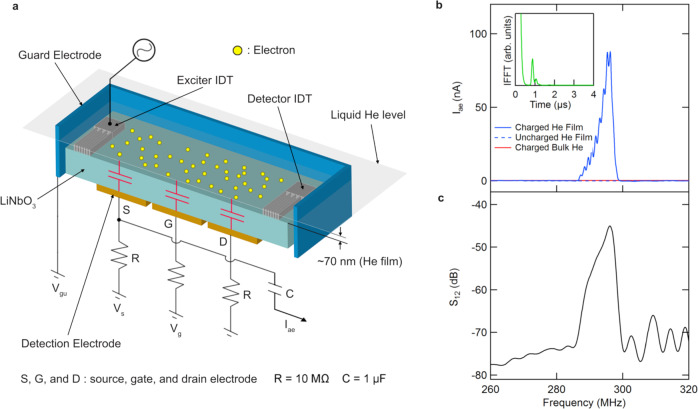
Schematic of the experimental setup and demonstration of continuous wave acoustoelectric transport of electrons on helium. **a** Cross-section view of the device for measuring SAW-driven transport of electrons on helium. Two opposing interdigitated transducers (IDTs) are used to excite and receive SAWs. A saturated superfluid ^4^He film is formed on the surface of the LiNbO_3_ piezo-substrate at *T* = 1.55 K. Thermionically emitted electrons are trapped above the surface of the superfluid film by applying positive bias voltages to three underlying electrodes arranged in a field-effect transistor (FET) configuration with a source (s), gate (g), and drain (d)^47^. Lateral confinement of the electron layer is achieved with a negative bias to the guard electrode positioned on the outside of the LiNbO_3_ substrate. **b** Measured acoustoelectric current *I*_ae_ of electrons on helium driven by a piezo-SAW as a function of frequency. For these measurements the FET electrode voltages were *V*_s_ = *V*_g_ = *V*_d_ = 40 V, corresponding to an electron density of *n* ≅ 0.8 × 10^9^ cm^−2^, and the guard was biased with − 3.2 V. Inset: inverse Fourier transform of the acoustoelectric current signal which reveals a peak at *t* ≈ 0.9 μs. This time scale corresponds to a SAW propagation distance of 3.2 mm, roughly the same as twice the distance between the launching IDT center and the near-edge of the LiNbO_3_ substrate, which implicates SAW reflections as responsible for the superimposed oscillations present on the acoustoelectric current peak. **c** Frequency dependence of the transmission coefficient (*S*_12_) of the SAW device demonstrating an expected resonance at 296 MHz.

Figure 1a shows a schematic of the device for producing and measuring SAW-driven acoustoelectric transport of electrons on helium (see Methods section). The electron systems floats above the surface of a thin superfluid film at a temperature *T* = 1.55 K that is supported by an underlying piezoelectric substrate made of highly-polished lithium niobate (LiNbO_3_). The superfluid film is estimated to be ≈70 nm thick based on the amount of helium admitted into the experimental cell as well as its open volume (see Supplemental Information Section 1). SAWs on the lithium niobate crystal are launched by applying a high-frequency voltage to an interdigitated transducer (IDT) on the surface of the lithium niobate and are directly detected using an opposing IDT (see Fig. 1a). The electron system is trapped and laterally confined on the surface of the superfluid film using voltages applied to electrodes beneath and around the piezo-substrate. The underlying electrodes also serve to capacitively detect the signal produced when the evanescent electric field of the SAW carries electrons along the surface of the superfluid in its propagation direction. With this device we are able to perform both continuous wave (cw) and gated acoustoelectric measurements. For continuous wave acoustoelectric transport measurements an amplitude modulated (AM) excitation signal was applied to the exciter IDT and the acoustoelectric current *I*_ae_ was measured using standard lock-in techniques. Time-of-flight measurements were performed by gating the SAW excitation signal on resonance (296 MHz) and the acoustoelectric current signal was collected using a fast, low-noise current preamplifier in tandem with a digital oscilloscope (see Methods section).

The acoustoelectric response for the case of cw SAW excitation is shown in Fig. 1b and coincides with the independently measured SAW resonance at 296 MHz (Fig. 1c). This acoustoelectric current *I*_ae_ is equivalent to the flux per unit time of electrons passing through the region above the underlying detection electrodes. No acoustoelectric current is observed without electrons present on the superfluid film (dashed blue trace in Fig. 1b) confirming that the signal arises from the electron layer floating on the helium surface. Moreover, we find that *I*_ae_ vanishes when the electrons are moved far away from the surface of the piezo-substrate by increasing the thickness of the superfluid layer (solid red trace in Fig. 1b) as would be expected given the evanescent character of the SAW potential above the piezoelectric surface. The acoustoelectric current signal shown in Fig. 1b also exhibits a periodic superimposed oscillation on top of the the main resonance. This corrugation in the acoustoelectric current is attributable to reflections of the SAW from the edge of the substrate, as is evident from the Fourier transform of the signal into the time domain (inset Fig. 1b). We also note that the frequency of surface capillary wave excitations of the helium is expected to be ~100 kHz at the SAW wavelength, far from the 296 MHz SAW resonance. More importantly, as described below our time-of-flight measurements clearly show that the acoustoelectrically transported electrons are propagating at the speed of the SAWs on lithium niobate (~10^3^ m/s) and not of the much slower helium capillary waves (~10 m/s), excluding their contribution to the measured current. Together these experiments confirm that the acoustoelectric current is generated by the transport of electrons on the superfluid surface via the SAW electric field extending up from the underlying piezo-substrate.

**Fig. 2:**
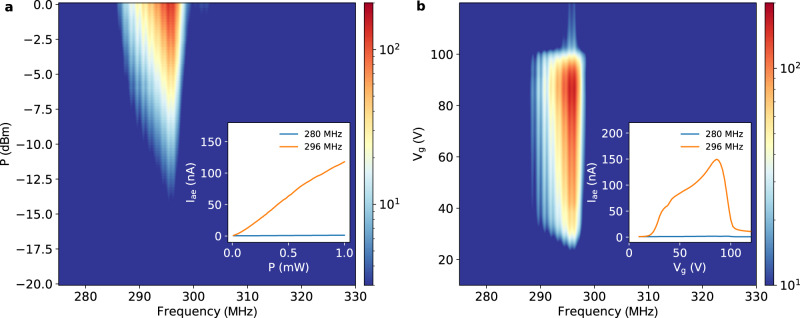
Power dependence and gate-tunability of the cw acoustoelectric effect with electrons on helium. **a** Acoustoelectric current *I*_ae_ measured as function of the SAW driving frequency and power. The inset shows that *I*_ae_ is linear in driving power when the frequency corresponds to the SAW resonance. **b** Demonstration of an acoustoelectric field-effect transistor (aFET) with electrons on helium. The inset shows line-cuts of *I*_ae_ both on- and off-resonance (orange and blue curves) with the SAW. These measurements were performed at *T* = 1.55 K and with *V*_s_ = *V*_d_ = 40 V, corresponding to *n* ≅ 0.8 × 10^9^ cm^−2^, and the guard electrode biased with −3.2 V.

The magnitude of this SAW-driven current depends on the extent to which the SAW potential can be screened by the electron system. In turn this will depend on the ratio of the plasma frequency, *ω*_p_ ≈ 15 GHz in our experiments, to the SAW frequency. In this regime, where *ω*_p_ ≫ *ω*_SAW_ the mobile electrons on the surface of the helium will quickly respond to screen the piezo-field of the SAW, leading to a traveling charge density wave that produces the low-frequency acoustoelectric current we observe (see Supplemental Information Section 2). A characteristic feature of this acoustoelectric transport in 2DESs is a linear dependence of the measured signal, in this case *I*_ae_, on the SAW intensity and hence excitation power (see Supplemental Information Section 2 and Eq. 11). Figure 2a shows the SAW power dependence of *I*_ae_ for electrons on helium. At the SAW resonance *I*_ae_ increases linearly with the RF input power consistent with this expected linear response (see inset Fig. 2a), which serves to further confirm the acoustoelectric origin of the measured signal from the system of electrons on helium.

Field effect control is crucial for the development of acoustoelectric devices^29,41–43^. In Fig. 2b we show a form of gate controlled SAW-driven electron transport. By tuning the gate bias voltage *V*_g_ we can turn *I*_ae_ ON and OFF, in effect creating an acoustoelectric field-effect transistor (aFET). When *V*_g_ is different from the source voltage *V*_s_, electrons dragged by the traveling SAW encounter an effective potential energy barrier *U*_eff_ = − *e*(*V*_g_ − *V*_s_) in the region above the gate. As shown in the orange trace of the Fig. 2b inset, for sufficiently small values of *V*_g_, acoustoelectric charge transport is blocked by a large positive *U*_eff_, which results in zero current. Upon increasing *V*_g_, electrons transported by the SAW are allowed to enter the region above the gate due to a decrease in *U*_eff_, which leads to a increase in *I*_ae_ at a threshold value of *V*_g_ determined by the overall areal electron density. Further increasing *V*_g_ eventually leads to a suppression of *I*_ae_ once the region above the source has been depleted of electrons (see Fig. 2b inset).

**Fig. 3:**
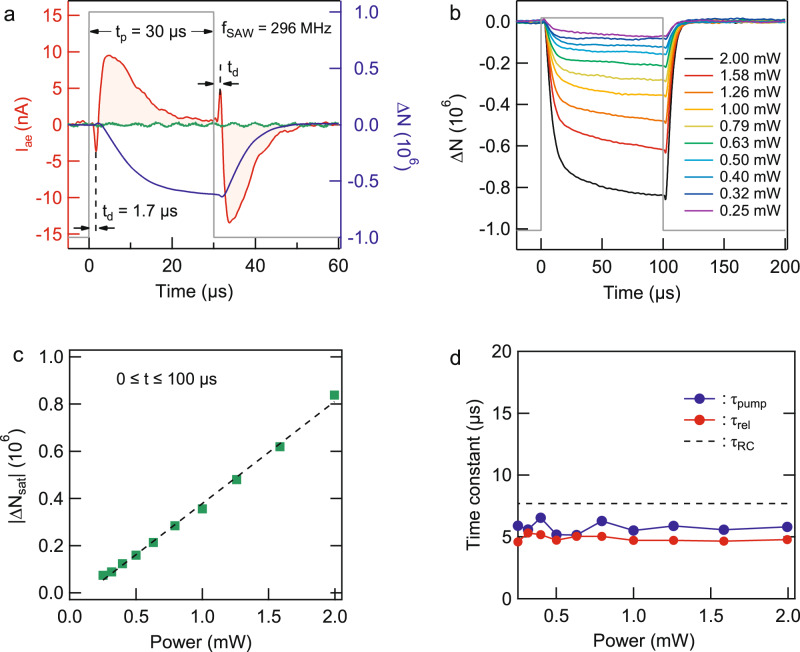
Time-of-flight acoustoelectric measurements of electrons on helium. **a** Measurement of *I*_ae_ (red curve) using a gated SAW (gray line) at a fixed RF power (0 dBm). These measurements were performed at the SAW resonance frequency (296 MHz). In these measurements the number of electrons above the detection electrode is *N* ≅ 0.4 × 10^9^. The blue trace is the calculated change in the number of electrons Δ*N*, which is obtained from the time integral of *I*_ae_ (red trace). We note that *I*_ae_ is absent when surface electrons are not present (green data trace), revealing that the measured current is induced by the SAW-electron interaction. **b** Δ*N* and **c** ∣Δ*N*_sat_∣ for a steady-state SAW signal at various values of the driving RF power. The dashed line is a guide to the eye. In these measurements the number of electrons is *N* ≅ 0.8 × 10^9^. **d** Dynamical response of two-dimensional electrons on helium to gated SAWs as a function of RF power with *N* ≅ 0.8 × 10^9^. The time constants *τ*_pump_ (*τ*_rel_) are those associated with the rising (falling) edge of the SAWs signal envelope and are determined from fitting *I*_ae_(*t*). The time constant *τ*_RC_ (dashed line) is that determined from low-frequency transport of electrons on helium.

An important step for future SAW-based experiments with electrons on helium, in particular those working with small numbers of electrons, is the ability to precisely control the number of SAW-transported electrons. This can be accomplished by gating the SAW in time, which we show in Fig. 3. In these time-of-flight measurements the SAW IDT is excited on resonance for a fixed period of time, *t*_p_, which launches a surface acoustic wave packet having a duration in time equal to *t*_p_. The envelope of the SAW-packet contains the high-frequency acoustoelectric field, which picks up electrons and carries them in the propagation direction of the SAW (see Methods section). For an areal electron density *n* the continuity equation for the acoustoelectric current density **j**_ae_,

1 $$\partial n/\partial t+\nabla \cdot {{\bf{j}}}_{\text{ae}}=0$$

predicts that an acoustoelectric current should appear once SAW-driven electrons flow past the boundary between the detecting electrodes. This behavior is shown in Fig. 3a for the case where *t*_p_ = 30 μs, where *I*_ae_ starts sharply increasing at a time *t*_d_ ≅ 1.7 μs after the SAW is launched. This delay in the onset of *I*_ae_ corresponds to the arrival time of the leading-edge of the gated SAW at the boundary above the detection electrode. The traveling SAW continues to drive electrons across the boundary, progressively building up an increasing charge imbalance in the electron layer. This charge imbalance produces an electric field that opposes the SAW-induced electron flow and leads to a decrease in *I*_ae_ as shown in Fig. 3a (red trace). Once the trailing edge of the gated SAW transits past the edge of the detection electrode (i.e. once *t* = *t*_p_ + *t*_d_) a current reappears but having the opposite polarity. In contrast to other 2DESs, these features are unique to electrons on helium and arise from the fact that this 2DES has a fixed total number of electrons. The SAW signal dynamically redistributes the electrons above the surface of the superfluid creating a non-equilibrium density, which then relaxes back to equilibrium after the passage of the SAW envelope.

We can quantitatively analyze this SAW-induced charge imbalance by calculating the time integral of *I*_ae_ to extract the change in the number of electrons ∣Δ*N*∣ above the detection electrode (blue trace Fig. 3a), which reaches a saturated steady-state value, ∣Δ*N*_sat_∣, in the limit of long SAW signal. Increasing the SAW power can be used to tune the magnitude of ∣Δ*N*_sat_∣ as shown in Fig. 3b, and in this regime the power dependence allows us to estimate the minimum number of transported electrons that can be detected for a given length of the SAW envelope (see Fig. 3c). For these measurements where *t*_p_ = 100 μs we find that for the lowest power as little as ∣Δ*N*/*N*∣ ≤ 10^−4^ of the number of electrons *N* ≅ 0.8 × 10^9^ can be transported within each 100-μs-long SAW envelope (see Supplemental Information Section 3). We anticipate that optimization of device geometry to include a combination of microchannel lateral confinement^24^ and single-electron transistor charge detection^44,45^ is a fruitful path forward for future experiments acoustoelectric experiments with even smaller numbers of electrons.

Finally, the gated SAW measurements allow us to extract information about the dynamical response of the many-electron system on helium by analyzing the build-up and relaxation of the SAW-induced charge pumping. Specifically, we fit the rising and falling edges of the acoustoelectric signal to an exponential and extract time constants *τ*_pump_ and *τ*_rel_, which are shown in Fig. 3d. In both cases, the time constants do not depend on the SAW power and we find *τ*_pump_ = 5.7 ± 0.1 μs and *τ*_rel_ = 4.9 ± 0.1 μs (see Supplemental Information Section 4). These time constants are ultimately determined by the underlying mechanisms that lead to electron scattering in the system. In the case of the present experiments, which were performed at *T* = 1.55 K, a strong scattering mechanism is the collision of electrons with helium vapor atoms above the superfluid surface. These scattering events occur at a frequency (≈18 GHz, see Supplemental Information Section 3) much larger than that of the SAW electric field (296 MHz). Therefore the time constants extracted from the SAW measurements should be similar in magnitude to the *RC* time constant expected from a transmission line modeling of the low-frequency transport measurements of the electron system, which we performed using the electrodes located underneath the LiNbO_3_ substrate (see Supplemental Information Section 3). Based on a transmission line analysis of these transport measurements we estimate that *τ*_RC_ = 7.7 μs, in reasonable agreement with our SAW measurements.

In conclusion, we have demonstrated the coupling of a system of electrons floating on the surface of superfluid helium to the evanescent field produced by a piezoelectric surface acoustic wave. This method allows us to perform controlled high-frequency acoustoelectric charge pumping within the electron system. These measurements also show that SAWs are a versatile tool for interrogating the dynamical processes in electrons on helium including, when extended to lower temperature, investigations of plasmon modes and ripplo-polaronic excitations of the Wigner solid^46^.

## Methods

The electrons on helium acoustoelectric device was measured inside a superfluid leak-tight copper cell, where a sheet of electrons produced from a tungsten filament were floating on top of ~70 nm thick liquid helium film. A YZ cut lithium niobate (LiNbO_3_) single crystalline chip with a length of 20 mm, a width of 10 mm, and a thickness of 0.5 mm was used as a substrate for both the superfluid film and SAW propagation as illustrated in Fig. 1a. A set of three rectangular electrodes (source, gate, and drain) located beneath the lithium niobate as well as a rectangular guard electrode outside of the substrate were used to trap and laterally confine electrons above the surface of the helium film by applying DC bias voltages to them. Each of the underlying trapping electrodes had a width of 4.95 mm and a length of 9 mm. On the surface of the LiNbO_3_ chip, two identical interdigitated transducers (IDT) consisting of 40 pairs of 3 μm wide fingers were patterned using standard optical lithography. The transducers were made of evaporated aluminum and had a thickness of ~70 nm and a width of 4 mm, which corresponds to the aperture of the SAW beam. By applying a high-frequency signal to the exciter IDT, SAWs are launched along the surface of the piezoelectric substrate toward the opposing detector IDT. The fundamental resonant frequency of our SAW device is calculated to be *ν* = *λ*/*v* = 291 MHz, dictated by the IDT finger periodicity, *λ* = 12 μm, and the speed of sound in YZ cut LiNbO_3_, *v* = 3488 m/s. We characterized the frequency response of the SAW used in our experiments using a vector network analyzer. Figure 1d shows the measured transmission coefficient *S*_12_ of the SAW delay line as function of frequency at *T* = 1.55 K. The resonance in the transmitted power at *ν* = 296 MHz is associated with the generation of SAW on the substrate. The slight difference between the expected resonant frequency and the measured value is likely due to piezo-crystal contraction at cryogenic temperature. With this experimental setup, acoustoelectric transport of electrons on helium was measured via capacitive coupling between electrons floating on the helium surface and a detection electrode beneath the lithium niobate. For continuous wave (cw) acoustoelectric transport measurements an amplitude modulated cw excitation signal having a modulation frequency of 20 kHz was applied to the exciter IDT using an Agilent 8648B RF signal generator and *I*_ae_ was measured using standard lock-in techniques. The time-of-flight measurements of *I*_ae_ were performed by gating the SAW excitation signal on resonance (296 MHz) and the acoustoelectric current signal was collected using an SR570 low-noise current preamplifier in tandem with a Tektronix DPO7054 digital oscilloscope. The acoustoelectric current waveform was averaged over 10,000 samples to improve signal to noise ratio in these measurements.

## Supplementary information

Supplementary Information

## Data Availability

The data that support the findings of this study are available from the corresponding authors upon reasonable request.
