# Supplementary material for: Piezoacoustics for precision control of electrons floating on helium
Source: Nat Commun. 2021 Jul 6;12:4150. doi: 10.1038/s41467-021-24452-7 (PMC8260748; doi:10.1038/s41467-021-24452-7)
Supplement: Supplementary file 1 — Supplementary Information [file 41467_2021_24452_MOESM1_ESM.pdf]

SUPPLEMENTAL INFORMATION

1. Superfluid film thickness

Fig. S1(a) shows the hermetically sealed copper cell used for the measurements described in the main manuscript. To fill the cell with superfluid ^{4}He, helium gas was supplied into the cell at $T \cong 1.55$ K through a capillary line at room temperature. The liquid helium volume admitted into the cell was determined by varying the pressure in a calibrated standard volume ($V = 260$ cc) located at room temperature. The thickness of a saturated helium film, d_0, can be estimated from [1],

$$\frac{\alpha}{{d_0}^4} = \rho g H, \tag{1}$$

where α is the van der Waals constant, g is the gravitational acceleration, ρ is the mass density of helium, and H is the distance from the $LiNbO_3$ substrate top surface down to the liquid helium surface in the reservoir volume inside the cell. We calculated H as function of the volume of helium admitted into the cell using 3D modeling of the cell open volume (see Fig. S1(b),(c)). For the thin film measurements reported in the manuscript, $H = 0.2$ mm, which corresponds to a thickness of the superfluid film, $d_0 \cong 77$ nm, from Eq.(1). Charging the helium film with electrons exerts an electronic pressure on the film [2] in addition to gravity and thus reduces the thickness of the charged helium film, d, which can be calculated from,

$$\frac{\alpha}{d^4} = \rho g H + p_{\mathrm{el}} = \frac{\alpha}{{d_0}^4} + 2\pi n^2 e^2, \tag{2}$$

where p_{el} is the electronic pressure, n is the electron density and e is the electron charge. We note that due to quantum electrodynamic effects, for thick films (> 60 nm) the van der Waals potential energy is proportional to $1/d^4$ rather than $1/d^3$ [3, 4]. In most of the acoustoelectric measurements reported in the manuscript, the areal electron density is estimated to be $n \simeq 0.8 \times 10^9$ cm^{-2} from field effect transistor (FET) measurements, which are described in the SI section 3. This yield a charged helium film thickness of $d \cong 72$ nm from Eq. (2).

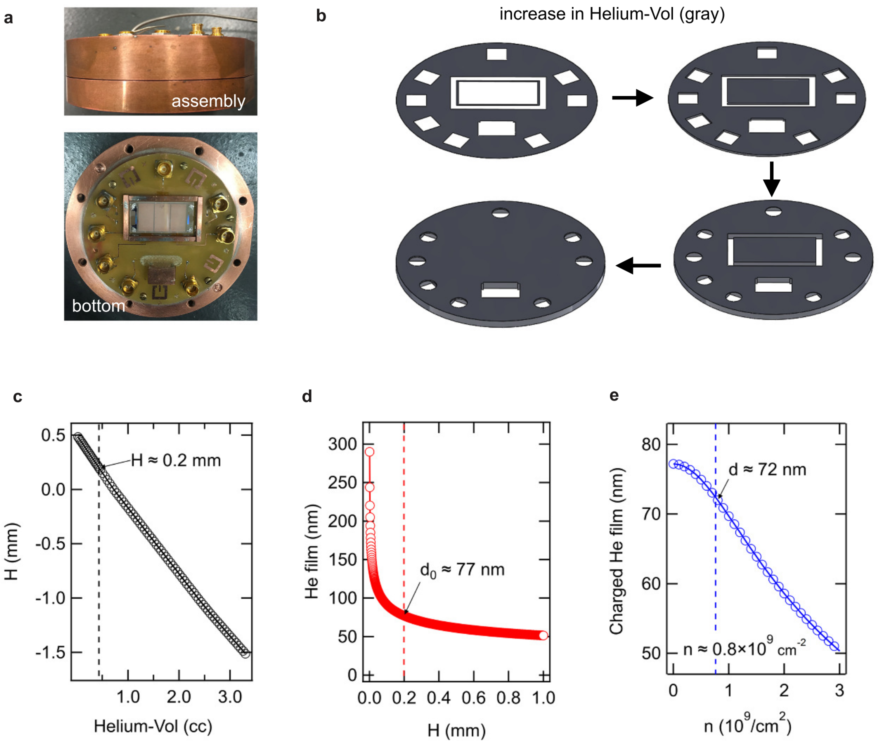

FIG. S1. **The experimental sample cell and helium film thickness determination.** **(a)** Photograph of the copper cell used for the SAW-based measurements of electrons on helium. The bottom portion of the cell contains a $LiNbO_3$ device (semi-transparent white) mounted on a printed circuit board **(b)** 3D CAD modeling of the liquid helium volume inside the experimental cell during filling with helium. **(c)** Calculation of H, the distance between the piezo-substrate top and the liquid helium level in the cell reservoir volume from the 3D CAD modeling of the open cell volume. The calculated value of H in the experiments is 0.2 mm for a helium vol. = 0.44 cc (vertical dashed black line). **(d)** Helium film thickness versus H before charging the film (see Eq. (1)). For an uncharged helium film $d_0 \approx 77$ nm (vertical dashed red line). **(e)** Charged helium film thickness versus electron density from Eq. (2). The charged helium film thickness is calculated to be $d \cong 72$ nm for an electron density of $n \cong 0.8 \times 10^9$ cm^{-2} (vertical dashed blue line).

2. SAW-2DES interaction and acoustoelectric transport

As a SAW propagates along the surface of a piezoelectric substrate, co-propagating electric fields can interact with a 2D electron system in close vicinity to the substrate surface. The electrons dynamically reorder themselves to accommodate the spatially and temporally varying SAW piezo-field. Via reciprocity, this interaction influences the propagation of SAW and leads to attenuation per unit length $\Gamma_{\rm el}$ and a shift of the SAW velocity $\Delta v/v_0$ given by [5]

$$\Gamma_{\rm el} = k\frac{K^2}{2}\frac{\sigma/\sigma_{\rm m}}{1+(\sigma/\sigma_{\rm m})^2} \tag{3}$$

$$\frac{\Delta v}{v_0} = \frac{v-v_0}{v_0} = \frac{K^2}{2}\frac{1}{1+(\sigma/\sigma_{\rm m})^2}, \tag{4}$$

where k is the wave number of the SAW, σ is the sheet conductivity of the 2DES, K^2 is an effective electromechanical coupling coefficient, $\sigma_{\rm m}$ is a characteristic conductivity that depends on material parameters, and v_0 is the SAW velocity on the free surface of the piezo-substrate in the absence of a 2DES.

Additionally, the ability of the SAW potential to trap and transfer electrons at the speed of sound in the substrate enables the generation of an acoustoelectric current ($I_{\rm ae}$) through the 2DES. The piezoelectric field induced by a SAW propagating along the x-axis is given by $E_{\rm p}(x,t) = Ae^{i(kx-\omega t)}$, where ω and k are the frequency and wavevector of the SAW and A is the amplitude fo the field. The dynamical response of the electron system acts to screen this potential to an extent dictated by the ratio of the plasma frequency to the SAW frequency. For an electron density is $n \sim 10^9$ cm^{-2} and the SAW wavevector $k \cong 0.5\times10^6$ m^{-1} the 2D plasma frequency is $(e^2n/2\varepsilon_0 m)^{1/2}k^{1/2}/2\pi \cong 15$ GHz, which is much larger than the SAW frequency $\omega_{\rm SAW}/2\pi \cong 0.3$ GHz. Thus, in our experiments the electrons on the surface of liquid helium effectively screen the piezoelectric field. In this regime the effective field becomes [5, 6]

$$E_{\rm eff}(x,t) = E_{\rm p}(x,t) + E_{\rm ind}(x,t) = \frac{E_{\rm p}(x,t)}{1+i(\sigma/\sigma_{\rm m})} \tag{5}$$

and is accompanied by a modulated surface electron density

$$n(x,t) = n_0 + \Delta n\, e^{i(kx-\omega t)} = n_0 + \Delta n(x,t), \tag{6}$$

where $E_{\rm ind}(x,t)$ is the screening field produced by the response of the electron system and n_0 is the equilibrium electron density. In the case where the SAW produces relatively small disturbances in the electron density (valid in this experiment), the modulated conductivity of the electron system may be expanded to first order

$$\sigma(x,t) = \sigma_0 + \frac{\partial\sigma}{\partial n}\,\Delta n(x,t) \tag{7}$$

with the local acoustoelectric current density given by

$$j_{\rm ae}(x,t) = \sigma E_{\rm eff}(x,t) = \sigma_0 E_{\rm eff}(x,t) + \frac{\partial\sigma}{\partial n}\;\Delta n(x,t)\;E_{\rm eff}(x,t). \tag{8}$$

From the continuity equation, $\partial_x\, j_{\rm ae} + \partial_t\,(-ne) = 0$, one can relate the oscillating charge density $\Delta n(x,t)$ to the effective field $E_{\rm eff}(x,t)$, i.e.

$$\Delta n(x,t) = -\frac{\sigma E_{\rm eff}(x,t)}{ev}, \tag{9}$$

where e and $v = \omega/k$ are electron charge and the SAW velocity. By substituting Eq. (9) into Eq. (8) $j_{\rm ae}$ can be seen to have both linear and quadratic dependence on the effective potential $E_{\rm eff}(x,t)$

$$j_{\rm ae}(x,t) = \sigma_0 E_{\rm eff}(x,t) - \frac{\partial\sigma}{\partial n}\frac{\sigma_0}{ev}E^2_{\rm eff}(x,t). \tag{10}$$

Our low-frequency (quasi-DC) measurements of the acoustoelectric current arise from the time average of this current density (i.e. $\langle j_{\rm ae}(x,t)\rangle_t$), in which only the quadratic term $E^2_{\rm eff}(x,t)$ contributes to the signal since $\langle E_{\rm eff}(x,t)\rangle_t = 0$. This results in the acoustoelectric current depending linearly the SAW intensity $I(x) = \sigma_0\langle E^2_{\rm eff}(x,t)\rangle_t/\Gamma_{\rm el}$, i.e.

$$\langle j_{\rm ae}(x,t)\rangle_t = \frac{\mu}{v}\Gamma_{\rm el}I(x), \tag{11}$$

where μ is electron mobility.

The intensity of the SAW can be expressed as $I(x) = (\alpha_{\rm L}P_{\rm in}e^{-\Gamma_{\rm tot}x})/w$, where $\alpha_{\rm L}$, $P_{\rm in}$, w, and $\Gamma_{\rm tot}$ denote the acoustoelectric conversion efficiency of the SAW-exciting IDT, the input RF power applied to the IDT, the SAW beam aperture, and the total attenuation per unit length of SAWs respectively. We note that the device used here is composed of 2D electrons floating above ≈ 70 nm thick helium film formed on a $LiNbO_3$ substrate. Such a helium film on $LiNbO_3$ is known to lead to additional SAW attenuation $\Gamma_{\rm He}$ [7]. Thus, the total attenuation of the SAW for the electrons on helium system is given by

$$\Gamma_{\rm tot} = \Gamma_{\rm el} + \Gamma_{\rm He}. \tag{12}$$

3. Characterization of the electron system on helium

Field effect transistor transport measurements

Fig. S2(a) and (b) illustrate the measurement set-up and the equivalent transmission line circuit model for the low-frequency transport properties of electrons on helium. After charging a helium film with electrons, we characterize the electron distribution with low-frequency field effect transistor (FET) operation of the device [8]. These measurements are done with a gate voltage $V_{\rm g}$ sweep at fixed source and drain voltage at a frequency of 60 kHz as shown in Fig. S2(c). For sufficiently small values of the gate voltage, $V_{\rm g} < V_{\rm th}$, all of the electrons are localized over the source and drain electrodes creating a depletion region above the gate, which leads to zero current through electron layer. At a threshold value of the gate $V_{\rm g} = V_{\rm th}$ an AC current onsets from electrons being attracted to the region above the gate. As $V_{\rm g}$ increases beyond $V_{\rm th}$, source-drain current quickly rises and reaches a maximum in a vicinity of uniform electron density ($V_{\rm s} = V_{\rm d} = V_{\rm g}$). After reaching its maximum value, the current begins decreasing and eventually vanishes as electron depletion over the source and drain electrode occurs. This ability to change the electron density above the individual electrodes with the application of a bias voltage enables the electrically switching ON and OFF of the acoustoelectric current in electrons on helium system (see Fig. 2(b) of the main manuscript).

Electron density

A homogeneous areal electron density n above all electrodes is achieved when $V_{\rm s} = V_{\rm g} = V_{\rm d}$ and the electron density can be calculated from the low-frequency FET transport data by measuring the difference ΔV between the case where the electron density is uniform and $V_{\rm th}$ (see Fig. S2(c)) [8],

$$n = \frac{2}{3}\frac{c_l}{e}\Delta V. \qquad (13)$$

Here, c_l is the capacitance per unit area between electron layer and underlying electrodes from geometrical configuration, which is given by

$$c_l = \epsilon_0 \left(\frac{1}{\frac{d_{\rm g}}{\epsilon} + \frac{d_{\rm s}}{\epsilon_{\rm s}} + \frac{d}{\epsilon}} \right) \cong \epsilon_0 \left(\frac{1}{\frac{d_{\rm g}}{\epsilon} + \frac{d_{\rm s}}{\epsilon_{\rm s}}} \right) \qquad \text{(for } d \ll d_{\rm g}\text{)}, \qquad (14)$$

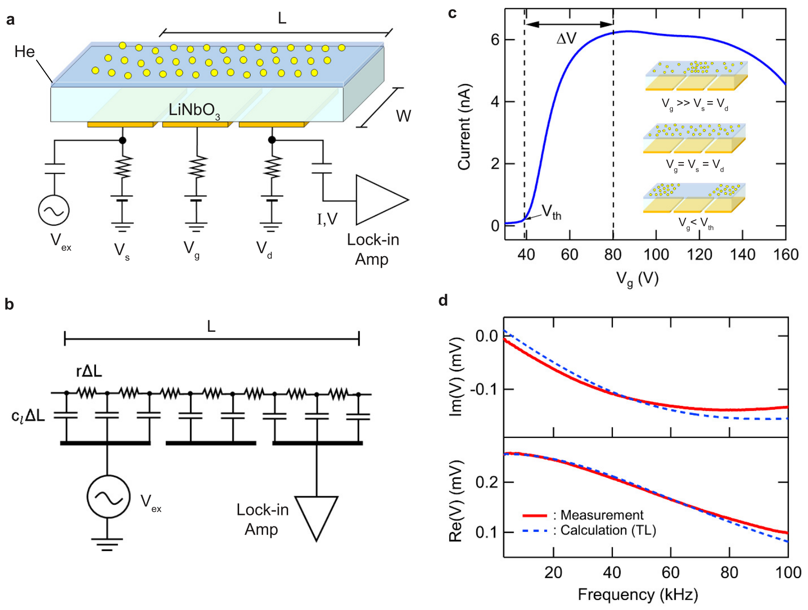

FIG. S2. **Experimental setup, circuit model, and measurement data for low-frequency transport of electrons on helium.** **(a)** Sketch of the electrons on helium device. A DC bias voltage is applied to the three underlying electrodes to trap electrons above helium surface. An AC excitation voltage ($V_{\mathrm{ex}} = 0.1$ V) applied to the source electrode induces a current through electrons on helium, which is capacitively detected using the drain electrode. **(b)** The equivalent transmission line circuit model for this device. The resistance of the electron sheet and the capacitance between this sheet and the three electrodes are spatially distributed per unit length, $r\Delta L$ and $c_l\Delta L$, where r and c_l represent the resistance per unit length and the capacitance per unit length. **(c)** Current-voltage characteristics for FET operation of the electrons on helium device. For this representative data, the source-drain current amplitude was measured during a gate voltage sweep with $V_{\mathrm{s}} = V_{\mathrm{d}} = 80$V. The insets show the corresponding electron density profiles over the electrodes for different values of V_{g}. A uniform areal density distribution is achieved at $V_{\mathrm{g}} = V_{\mathrm{s}} = V_{\mathrm{d}}$. **(d)** Frequency dependence of the transport characteristics of the system of electrons on helium. The red and blue traces represent the experimental data and the calculation based on the transmission line model respectively. For these measurement, all three electrodes were biased with 80 V ($V_{\mathrm{g}} = V_{\mathrm{s}} = V_{\mathrm{d}} = 80$ V).

where ϵ_0 is the vacuum permittivity, $\epsilon = 1.057$ is the dielectric constant of helium, $\epsilon_{\rm s} \cong 35$ is the effective dielectric constant of the $LiNbO_3$, $d_{\rm g} \cong 70$ μm is the gap between the $LiNbO_3$ substrate and the bottom electrodes, $d_{\rm s} = 0.5$ mm is the thickness of the $LiNbO_3$ substrate, and d is the charged helium film thickness. The effective dielectric constant $\epsilon_{\rm s}$ for $LiNbO_3$ is given by $\epsilon_{\rm s} = \sqrt{\epsilon_{11}\epsilon_{33} - \epsilon_{13}^2}$ where ϵ_{11}, ϵ_{33}, and ϵ_{13} are the dielectric tensor element of $LiNbO_3$ at constant stress [9]. These dielectric elements are given in Ref. [10] as $\epsilon_{11} = 44.3$, $\epsilon_{33} = 27.6$, and $\epsilon_{13} = 0$. With $c_l \cong 1.08 \times 10^{-7}$ F/m^2 obtained from Eq. (14), the uniform areal electron density is found to be $n \simeq 0.8 \times 10^9$ cm^{-2} for most of acoustoelectric measurements reported in this manuscript. For the power dependent pulsed SAW measurements (Fig. 3(b)-(d)), the value of n is about 1.9 $\times 10^9$ cm^{-2}. We note that in the gate-tunable acoustoelectric measurements shown in Fig. 2(b), n is no longer a uniform density but rather varies as a function of $V_{\rm g}$, however the total number of electrons over the device is fixed.

Electron mobility

Using the electrodes located beneath the $LiNbO_3$ substrate we are able to perform low-frequency transport measurements of the system of electrons on the helium thin film. The conductivity σ of electrons on helium from our low-frequency transport measurements can be determined by fitting the frequency response measurement to a RC transmission line model [11, 12] with σ as fitting parameter [8]. Fig. S2(d) shows this frequency response for the representative case where $V_{\rm s} = V_{\rm g} = V_{\rm d} = 80$ V, which shows good agreement with this model fit. The value of the conductivity from this fit is $\sigma \cong 1.58 \times 10^{-6}$ Ω^{-1}. The mobility μ of electrons on helium is then calculated based on a Drude model analysis ($\sigma = ne\mu$). For an electron density of $n = 1.9 \times 10^9$ cm^{-2} the mobility is $\mu \cong 5.3 \times 10^3$ cm^2/Vs with $d \cong 60$ nm. This mobility is roughly two orders of magnitude less than the mobility of electrons on bulk ^{4}He at the same temperature [13]. Such a low mobility for electrons on a thin superfluid film is consistent with previous measurements and can be explained by the close proximity of the electron layer to the underlying substrate [14].

RC delay time constant

The RC delay time constant, $\tau_{\rm RC}$, introduced in the manuscript is calculated based on the transmission line modeling described above (see Fig. S2(b)) and the Elmore delay model [15], which is encapsulated by the following equation

$$\tau_{\rm RC} = r c_l L^2 \frac{1+N}{2N} \cong \frac{R_{\rm tot} C_{\rm tot}}{2} \quad \text{(for large N)}, \tag{15}$$

where r and c_l are the resistance per unit length and the capacitance per unit length of the electron system and L, $R_{\rm tot}$, $C_{\rm tot}$, and N represent the total length, the total resistance, the total capacitance, and the number of the node in the entire transmission line. $R_{\rm tot} = \sigma^{-1} W^{-1} L$ and $C_{\rm tot} = c\, W L$ are approximately 1.06 MΩ and 14.6 pF for $W = 9$ mm (the width of the electrodes in the SAW device) and $L = 15$ mm, which yields $\tau_{\rm RC} \approx 7.7$ μs in the low-frequency transport regime.

Electron collision rate with helium vapor atoms

At a temperature $T = 1.55$ K, ^{4}He vapor atoms are a strong source of electron scattering. The collision rate $1/\tau_{\rm He}$ is determined by

$$\frac{1}{\tau_{\rm He}} = \frac{e}{\mu m^*} \tag{16}$$

with the effective electron mass $m^* = m_{\rm e}$ (bare electron mass) and the electron mobility $\mu \cong 1 \times 10^5$ cm^2/Vs [13]. This yields $1/\tau_{\rm He} \simeq 18$ GHz.

4. Determination of SAW charge pumping time constants

The time constants for SAW-driven charge pumping ($\tau_{\rm pump}$) and subsequent relaxation ($\tau_{\rm rel}$) for different RF power was determined from an exponential curve fitting of the acoustoelectric current ($I_{\rm ae}$) data as shown in Fig. S3 and resulting values are tabulated in the table below.

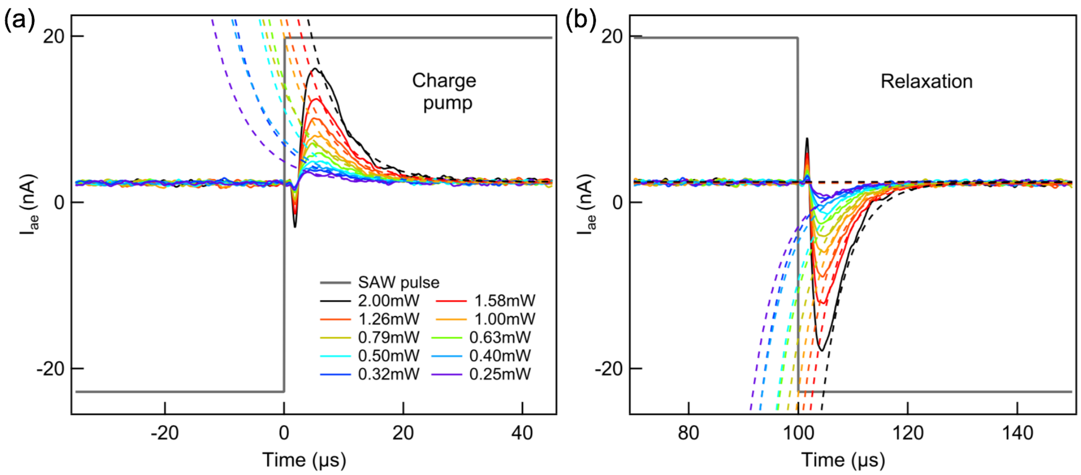

FIG. S3. **Determination of acoustoelectric time constants**. These time constants were determined from exponential fits to the acoustoelectric current data during **(a)** SAW excitation (charge pumping) and **(b)** relaxation after the SAW drive was removed.

RF (mW)	τ_{pump} (μs)	τ_{rel} (μs)
0.25	5.9	4.6
0.32	5.6	5.3
0.40	6.5	5.2
0.50	5.2	4.7
0.63	5.2	5.0
0.79	6.3	5.0
1.00	5.5	4.7
1.26	5.9	4.7
1.58	5.6	4.7
2.00	5.8	4.8

TABLE I. **Acoustoelectric time constants**. Values of time constants obtained from the exponential curve fits in Fig. S3 with varying SAW excitation power.

SUPPLEMENTARY REFERENCES

[1] Pobell, F. *Matter and methods at low temperatures*, vol. 2 (Springer, 2007).

[2] Leiderer, P. Electrons at the surface of quantum systems. *Journal of Low Temperature Physics* **87**, 247–278 (1992).

[3] Takita, M. *et al. Electrons on superfluid helium: towards single electron control.* Ph.D. thesis, Princeton University (2015).

[4] Klier, J., Schletterer, F., Leiderer, P. & Shikin, V. Equilibrium helium film in the thick-film limit. *Low Temperature Physics* **29**, 716–719 (2003).

[5] Wixforth, A. *et al.* Surface acoustic waves on GaAs/AlGaAs heterostructures. *Physical Review B* **40**, 7874 (1989).

[6] Esslinger, A. *et al.* Ultrasonic approach to the integer and fractional quantum Hall effect. *Surface Science* **305**, 83–86 (1994). URL `https://www.sciencedirect.com/science/article/pii/003960289490863X`.

[7] Byeon, H. *et al.* Anomalous attenuation of piezoacoustic surface waves by liquid helium thin films. *Journal of Low Temperature Physics* **195** (2019). URL `https://doi.org/10.1007/s10909-018-02115-0`.

[8] Nasyedkin, K. *et al.* Unconventional field-effect transistor composed of electrons floating on liquid helium. *Journal of Physics: Condensed Matter* **30**, 465501 (2018).

[9] Müller, C. *et al.* Surface acoustic wave investigations of the metal-to-insulator transition of V_2O_3 thin films on lithium niobate. *Journal of applied physics* **98**, 084111 (2005).

[10] Jazbinšek, M. & Zgonik, M. Material tensor parameters of $LiNbO_3$ relevant for electro-and elasto-optics. *Applied Physics B* **74**, 407–414 (2002).

[11] Lea, M., Stone, A., Fozooni, P. & Frost, J. The ac response of a 2-d electron gas on liquid helium in a magnetic field. *Journal of low temperature physics* **85**, 67–89 (1991).

[12] Mehrotra, R. & Dahm, A. Analysis of the Sommer technique for measurement of the mobility for charges in two dimensions. *Journal of low temperature physics* **67**, 115–121 (1987).

[13] Iye, Y. Mobility of electrons in the surface state of liquid helium. *Journal of Low Temperature Physics* **40**, 441–451 (1980).

[14] Shikin, V., Klier, J., Doicescu, I., Würl, A. & Leiderer, P. Dip problem of the electron mobility on a thin helium film. *Physical Review B* **64**, 073401 (2001).

[15] Rabaey, J. M., Chandrakasan, A. & Nikolic, B. *Digital Integrated Circuit Design a Design Perspective* (Prentice Hall, 2002), 2nd edn.
